# Dr. A. W. Calhoun

**Published:** 1872-02

**Authors:** 


### DR. A. W. CALHOUN.

We again present our readers an article from our accomplished correspondent Dr. Calhoun, of this State, now perfecting himself in his medical education, in Berlin, Prussia. His articles give evidence of great energy and study in accomplishing his design. He certainly gives promise of a brilliant future in his profession.

We have still another article of Dr. Calhoun's on hand, which will appear next month. We trust he will continue to give our readers the benefit of *his* observations at the great medical centre of Europe. We also thank him for his kind efforts in securing for us an exchange, and trust he will have it *properly* directed, as the copies sent by him have not yet come to hand.

